# In meeting the increasing demands for total knee arthroplasty, can we achieve high levels of quality care in a small community hospital? A mixed-methods study

**DOI:** 10.3389/fsurg.2023.998301

**Published:** 2023-02-14

**Authors:** Ethan B. Sanders, Johanna S. Dobransky, Brian P. Chen, Andrew W. Bodrogi, on behalf of Ottawa Arthroplasty Group, Paul E. Beaulé, Stéphane Poitras

**Affiliations:** ^1^ Division of Orthopaedic Surgery, The Ottawa Hospital, Ottawa, ON, Canada; ^2^ School of Rehabilitation, University of Ottawa, Ottawa, ON, Canada

**Keywords:** total knee arthroplasty, Canada, length of stay, small community hospital, tertiary care hospital

## Abstract

**Purpose:**

Small community hospitals (SCHs) help meet the demand for total knee arthroplasty (TKA). This mixed-methods study compares outcomes and analyses of environmental differences following TKA at a SCH and a tertiary care hospital (TCH).

**Methods:**

*Quantitative*: A retrospective review of 352 propensity-matched primary TKA procedures at both a SCH and a TCH, based on age, body mass index, and American Society of Anesthesiologists class, was completed. Groups were compared by length of stay (LOS), 90-day emergency department visits, 90-day readmissions, reoperations, and mortality. *Qualitative*: Based on the Theoretical Domains Framework, seven prospective semistructured interviews were performed. Interview transcripts were coded and belief statements were generated and summarized by two reviewers. Discrepancies were resolved by a third reviewer.

**Results:**

*Quantitative*: The average LOS for the SCH was significantly shorter than that for the TCH (2.0 ± 0.2 vs. 3.6 ± 2.7 days; *p* < 0.001), a difference that persisted following a subgroup analysis of ASA I/II patients (2.0 ± 0.2 vs. 3.2 ± 2.2; *p* < 0.001). There were no significant differences in other outcomes. *Qualitative*: The main themes that revolved around a higher case load for physiotherapy at the TCH resulted in patients waiting longer to be mobilized after surgery. Patient disposition also affected their discharge rates.

**Conclusion:**

Given the increasing demand for TKA, the SCH represents a viable option to increase capacity, while reducing LOS. Future directions to reduce LOS include addressing social barriers to discharge and patient prioritization for assessment by allied health services. When TKA is performed by the same set of surgeons, the SCH provides quality care with a shorter LOS and comparable with urban hospitals, and this can be attributed to the differences in resource utilization in the two hospital settings.

## Introduction

Total knee arthroplasty (TKA) is an efficient treatment for moderate to severe osteoarthritis (1–4). More than 70,000 knee replacements were performed in Canada between the years 2017 and 2018, which represents an increase of 17% over the last 5 years, with the demand expected to grow by 673% by 2030 (5, 6). There is also an increasing demand in younger patients, with 55% of TKA procedures projected to be implanted in patients younger than 65 years of age by 2030 (6). Keeping up with this level of demand will create a strain on the healthcare system. Given that a TKA costs a hospital an average of $9,339 CAD per patient, cost-reduction methods such as decreasing the length of stay (LOS) have become a priority for hospital administrators and provincial ministries of health (7, 8). This is especially relevant in a pandemic situation, during which small community hospitals (SCHs) could be used for dealing with higher patient volumes due to a higher prevalence of COVID-19.

In the United States, recent studies have demonstrated that patients undergoing TKA at an Orthopaedic Speciality Hospital (OSH) have a significantly shorter LOS, with no difference in 90-day readmissions or patient satisfaction compared with a TKA done at Tertiary Care Hospitals (TCHs) (7, 9–11). Length of stay, in particular, is a major driver of cost (12). This finding has its echo in shoulder arthroplasty and hip arthroplasty as well, demonstrating consistency across joints (13, 14). A major concern with reducing LOS is the effect on readmission rates. However, studies have demonstrated equivalent to improved perioperative outcomes at OSHs, with one study demonstrating a significantly lower rate of readmissions (11, 13). Canada has numerous SCHs, which are defined as hospitals that perform a little under 2,700 acute care and day surgeries for any two of the three immediately preceding years (15). While several studies have demonstrated that patients undergoing TKA at OSHs have fewer comorbid conditions, the impact on LOS in SCHs is still uncertain (13, 16).

Limited evidence is available comparing patient outcome data between SCH and TCH for TKA. The purpose of this study is twofold: (1) To compare readmissions, reoperations, and LOS between an SCH and a TCH in patients undergoing TKA, and (2) To better understand the environmental contexts in each institution contributing to differences between health outcomes.

## Materials and methods

This was a mixed-methods study involving a retrospective chart review of patient data, followed by semistructured interviews with healthcare providers at an SCH and a TCH. This study was approved under our institutional quality improvement portfolio.

### Quantitative study

A retrospective chart review of all primary TKA procedures performed at a SCH between January 2014 and December 2015 (*n* = 565) was completed. Patients undergoing the procedures were propensity-matched to patients who underwent a primary TKA at a TCH over the same period, on the basis of body mass index (BMI), age, and comorbidities [American Society of Anesthesiologists (ASA) class]. As the procedures were performed at the SCH only from Monday to Thursday due to a lack of inpatient services over the weekend, in the TCH cohort, patients operated only on these week days were included, in order to reduce the need for making available inpatient services over the weekend. These matched groups were then compared by LOS, 90-day emergency department (ED) visits, 90-day readmissions, reoperations, and mortality. All procedures were performed by a group of five fellowship-trained arthroplasty surgeons who operated at both types of hospitals. Postoperative protocols at the SCH were developed on the basis of the previously established protocols at the TCH. All patients operated on at the SCH were screened by applying the anesthesia exclusion criteria (Appendix A).

A power analysis was carried out, and it revealed with the use of an *α*-value of 0.05, a power of 0.8, and an effect size of 0.58 based on a previous study (7) that the sample size should be *n* = 47 in each group to detect significant differences in LOS. The independent samples *t*-test and chi-square tests were used to compare continuous and categorical variables, respectively. Significance was set at a *p*-value <0.05. SPSS version 26.0 (IBM, Armonk, NY, United States) was used for all quantitative analyses.

### Qualitative study

A semistructured interview guide was constructed on the basis of the Theoretical Domains Framework (TDF) to understand the factors influencing outcomes at both types of institutions (17–19). The TDF is based on a synthesis of 33 behavioral change theories organized into 14 domains (17–19). We used a purposive sampling technique to recruit healthcare providers well versed in the care of TKA patients at both the SCH and the TCH. We conducted 45-min interviews with three surgeons who had worked at both sites, one nurse from the TCH, and three physiotherapists (PTs), two from the TCH and one from the SCH, until we reached a level of saturation.

All interviews were digitally recorded and transcribed verbatim by using an official transcription service. The transcripts were then analyzed using a five-step process, adapted from a validated six-step technique as follows: (1) The transcripts were independently coded using the 14 domains in the TDF; (2) Belief statements were developed for each quote and similar statements were merged into one statement; (3) Shorter themes were created on the basis of the merged belief statements; (4) Themes were then merged into broader categories; (5) Themes were categorized in order of frequency stated (20). A third researcher was available to resolve any discrepancy.

## Results

### Quantitative study

Propensity score matching resulted in 352 patients per group, for a total of 704 patients, 261 male and 443 female. The overall mean age was 66.6 ± 9.5 years old, a BMI of 30.8 ± 5.6 kg/m^2^, and 383 ASA grades I/II and 321 ASA grades III/IV (Table 1). The average LOS for the SCH was significantly shorter than that for the TCH (2.0 ± 0.2 vs. 3.6 ± 2.7 days; *p* < 0.001). There were no significant differences in the rates of surgery-related 90-day readmissions (SCH 1.4% vs. TCH 1.1%; *p* = 0.7) or surgery-related 90-day ED visits (SCH 5.1% vs. TCH 4.0%; *p* = 0.5). The reoperation rates were 0.57% and 0.85% for the SCH and the TCH, respectively (*p* = 0.7) (Table 2). A subgroup analysis of patients with only ASA 1 or 2 continued to show a significantly shorter LOS (SCH 2.0 ± 0.2 vs. TCH 3.2 ± 2.2 days; *p* < 0.001) with no differences in adverse outcomes. Among ASA 3 or 4 patients, there was also a significantly shorter LOS at the SCH (SCH 2.0 ± 0.3 vs. TCH 4.1 ± 3.1 days; *p* < 0.001).

**Table 1:** Demographic variables of propensity-matched groups.

Variable	TCH (*n* = 352)	SCH (*n* = 352)	*p*-value
Age (mean ± SD)	66.6 ± 10.6	66.5 ± 8.19	0.9
Sex (male:female)	121:231	140:212	0.138
BMI (mean ± SD)	30.8 ± 6.16	30.8 ± 5.04	0.9
**ASA grade (count)**			
1 and 2	192	191	—

TCH, tertiary care hospital; SCH, small community hospital; ASA, American Society of Anesthesiologists; BMI, body mass index.

**Table 2:** Comparison of TCHs and SCHs on outcomes of interest.

Outcome of interest	TCH	SCH	*p*-value
Length of stay in days (mean ± SD)	3.60 ± 2.69	1.96 ± 0.24	<0.001*
N 90-day readmissions—surgery related (%)	4 (1.14%)	5 (1.42%)	0.7
N 90-day ED visits—surgery related (%)	14 (3.98%)	18 (5.11%)	0.5
N reoperations (%)	3 (0.85%)	2 (0.57%)	0.7

TCH, tertiary care hospital; SCH, small community hospital; ED, emergency department.

* Significance set at *p* < 0.05.

### Qualitative study

As a result of the significant difference in LOS between both sites, our interviews focused on better understanding the factors influencing this outcome. By the time of completion of the interviews, saturation had been achieved. The most salient themes centered around environmental context/resources and social influences (Table 3). Patients were mobilized sooner at the SCH than at the TCH because there were fewer patients per PT (*n* = 31 times stated), allowing the staff to provide more timely care. There was a higher patient case load for PTs at the TCH, which resulted in patients waiting longer to be mobilized after surgery. In addition, at the TCH, patient overflow from the orthopedic floor to the units resulted in non-orthopedic PT coverage.

**Table 3:** List of the most salient themes identified through seven qualitative interviews (identified by at least two different interviewees).

Theme	Number of times stated	Stated by (the number of different healthcare providers)	Domain
Patient-specific factors affect the ability to meet discharge criteria	36	Surgeons (3), Physio at the TCH (2), Physio at the SCH (1), Clinical Manager at the TCH (1)	Environment and context (25), knowledge (4), memory, attention and decision making (6), social influences (1)
Physio availability affects discharge	31	Surgeons (2), Physio at the TCH (2), Physio at the SCH (1), Clinical Manager at the TCH (1)	Beliefs about capabilities (2), environment and context (23), goals (1), knowledge (1), memory, attention, and decision making (2), social professional role and identity (2)
Case-mix differences (Complex patients done at the TCH)	26	Surgeons (3), Clinical Manager at the TCH (1), Physio at the TCH (1)	Environment and context (18), Social/professional role and identity (1), Knowledge (5), Memory, decision making (2)
Environmental differences between sites	26	Surgeons (3), Physio at the TCH (2), Physio at the SCH (1), Clinical Manager at the TCH (1)	Behavioral regulation (1), beliefs about capabilities (1), environment and context (17), goals (1), knowledge (2), memory, attention, and decision processes (1), skills (1), social/professional role and identity (2)
Communication inconsistencies between healthcare providers and patients	19	Surgeons (2), Physio (1)	Beliefs about capabilities (1), environment and context (2), memory, attention, and decision making (1), social influences (15)
Incentives can reduce LOS	19	Surgeons (3), Physio at the TCH (1), Clinical Manager at the TCH (1)	Emotion (3), environment and context (2), goals (3), reinforcements (11)
Collaborative team approach	16	Surgeons (2), Physio at the TCH (2), Physio at the SCH (1), Clinical Manager at the TCH (1)	Memory, attention, and decision making (3), social influences (7), social/professional role and identity (1), emotion (1), environment and context (3), behavioral regulation (1)
Ownership over discharge	14	Surgeons (3), Physio at the TCH (1), Physio at the SCH (1), Clinical Manager at the TCH (1)	Beliefs about capabilities (1), environment and context (1), social influences (2), social/professional role and identity (10)
Inconsistent discharge instructions	13	Surgeons (1), Physio at the TCH (1), Physio at the SCH (1), Clinical Manager at the TCH (1)	Behavioral regulation (2), environment and context (3), memory, attention, and decision processes (3), social influences (5)
Early planning for discharge improves LOS	12	Surgeons (2), Physio at the TCH (1), Clinical Manager at the TCH (1)	Beliefs about capabilities (1), environment and context (6), knowledge (2), social influences (3)
Symptom management delays discharge	10	Surgeon (1), Physio at the SCH (1), Clinical Manager at the TCH (1)	Environment and context (4), knowledge (3), memory, attention, and decision making (3)
Benefits of reducing LOS	8	Surgeons (2), physio at the SCH (1)	Beliefs about consequences
Consequences of reduced LOS	8	Surgeons (2), Physio at the SCH (1), Clinical Manager at the TCH (1)	Beliefs about consequences (7), environment and context (1)
Belief that no further reduction in LOS is possible	6	Surgeons (3), Physio at the TCH (1)	Goals (1), intentions (1), beliefs about capabilities (4)
Nurse availability affects discharge	6	Surgeons (2), Physio at the TCH (1), Clinical Manager at the TCH (1)	Environment and context (6)
Availability of outpatient care	4	Surgeon (1), physio at the TCH (3)	Social influences (1), environmental context (2), knowledge (1)
LOS reduction is realistic.	4	Surgeon (1), Physio at the SCH (1), Clinical Manager at the TCH (1)	Beliefs (2), Optimism (2)
No incentive to change practice	4	Surgeon (1), Physio at the TCH (1)	Reinforcements (2), intentions (2)
Prioritizing patients likely for early discharge	3	Surgeon (1), Physio at the TCH (1)	Behavioral regulation (1), goals (1), beliefs about capabilities (1)

TCH, tertiary care hospital; SCH, small community hospital; LOS, length of stay.

*“The patients here (TCH) will go off ward if orthopedics is full so they get up on urology ward or general surgery ward or medicine ward and then they don’t get the therapy. It’s not even the ortho therapist.”*

Workflow and case-mix differences between sites (*n* = 26 times stated) were noted as a factor affecting LOS. This relates to the streamlined nature of care received at the SCH in which PTs on the orthopedics floor handle only arthroplasty patients.

*“that unit ward at (SCH) is spring-loaded for a one-day discharge, two exceptionally and so that’s all they know. That is all they do. That is all they know. Here (TCH), we’ve got a whole slew of different patients with trauma and everything else.”*

Another key theme identified was patient-specific factors influencing discharge (*n* = 36 times stated). Social issues such as patient disposition and resources were common themes affecting the individual patient’s ability to get discharged.

*“So, I think that there’s a greater proportion of social support at (SCH), because they got to get somebody to drive them out. So, therefore that person drives them back.”*

Considering the context within the healthcare team, six of seven interviewees (*n* = 14 times stated) identified a lack of ownership over discharge at the TCH, as each healthcare practitioner felt another had a greater role in determining the date of discharge. This situation was in contrast to the SCH, where the physician controlled the discharge process. This led to inconsistent discharge rates tied to communication (*n* = 13 times stated).

*“anyone who says to the patient I think you need to stay another day. As soon as that’s mentioned anywhere along the way, (the discharge plan) all falls apart.”*

When considering the motivation and readiness for behavioral change, the interviewees demonstrated varied opinions when considering a potential decrease in LOS at the TCH.

*“it reduces costs and increases flow-through of patients, freeing up beds, (increasing) access for emergency patients. There is more satisfaction in being home (for the patients)”*

*“the negative would be sending them home too quickly and having a medical events. That’s the tightrope we walk on”*

## Discussion

Providing quality care at a lower cost has been a major focus of both private and publicly funded healthcare systems (21). The introduction of bundle care is a prime example where the provider is responsible for the whole episode of care, that is, both inpatient and outpatient care within 90 days of the surgical intervention. Early experience with this model has led to a decrease in overall healthcare costs, while maintaining a high level of quality (22). While Canada is in its early days of bundle care, hospital bed occupancy and limited resources for highly specialized services can make meeting the increasing demand of primary hip and knee replacements a challenge. This is certainly true for TKA, whose incidence is growing at a higher rate than total hip arthroplasty (5). Hence, the importance of developing innovative centers of care such as SCHs and outpatient joints to improve the delivery of care, while minimizing the impact of such care on healthcare resources cannot be underestimated (23). Such care has assumed significance especially during the period of the COVID-19 pandemic considering the backlog of joint replacements created, as well as competing resources for higher-acuity surgeries such as cancer at TCHs (24). Therefore, creating new pathways for the delivery of care becomes critical to providing quality care for arthroplasty patients. After propensity matching of patients for known predictors of LOS, our study showed that TKA procedures at the SCH are safe and effective with significantly shorter LOS and equivalent ED visits, readmissions, and adverse event outcomes.

Strategies to mitigate increasing healthcare costs have been a subject of significant interest. A study conducted by Lovse et al., highlights that TKA does not follow the Pareto principle, with all patients regardless of complexity contributing to increased costs (12). This emphasizes the need to decrease LOS, a major contributor to direct healthcare costs for TKA for all patients (25). The SCH avoids treating patients with severe comorbidities. However, when performing a subgroup analysis on ASA grade, it was found that LOS was still significantly different for both grade groups.

By propensity matching and controlling for surgery-related variables, we were able to isolate the healthcare team and the hospital environment in terms of their roles related to LOS. Respondents attributed the lower LOS at the SCH primarily to resource utilization differences, with PT availability being a common theme. There does, however, exist some confusion with control over patient discharge. While physicians ultimately control discharge from hospital, surgeons feel that members of the allied health team often delay discharge on the ground that patients are not yet ready for it. This is largely related to a lack of communication among providers, as the parameters of pain control, mobility, and toleration of oral intake are assessed by numerous providers. Without this communication, the message to the patient can be less direct and therefore confusing. However, a balance always exists between cost reduction resulting from changes in LOS and quality of care. The SCH’s orthopedic unit seems to provide the optimal balance, providing good patient outcomes and lower cost of care.

The advantages of SCHs over TCHs may be attributed to the heterogenous population of orthopedic patients at TCHs, where only 6 of 37 beds (16%) are reserved for arthroplasty patients, and thus, there is a significant variation in postoperative and discharge plans between patients. The healthcare team is responsible for patient treatment across all adult orthopedic subspecialities at TCHs, including emergent and urgent cases, often requiring higher levels of care. Patients are often prioritized on the basis of need, with the most severe cases getting the most attention. However, at SCHs, a smaller ward dedicated to elective arthroplasty patients can provide streamlined care.

The findings of our study are similar to those of recent studies demonstrating differences in LOS and similar outcomes (7, 9). One common criticism of the OSH is selection bias, because generally, only healthier patients are admitted at such hospitals. Also, the selection criteria for admission, which are based on the available resources, are strict so as to handle only those patients who have complex medical conditions (11). To control for this, in our study, patients were matched by previously reported outcome-influencing preoperative factors. However, our study still included a high percentage (46%) of patients with ASA III/IV grade surgeries performed at the SCH with a lower LOS and no difference in terms of adverse events.

We suggest that our study is both relevant and generalizable to the Canadian context, when considering the utility of the SCH in the management of the growing demands for TKA. However, this mixed-methods study is not without limitations. Considering the retrospective part of the study, there are inherent limitations pertaining to its design. Furthermore, despite our reaching a saturation level through interviews, it was possible that the views of those interviewed differed from those who did not participate, and that a less homogenous group, that is, different surgeons operating at the SCH, may not provide equivalent outcomes. Given that the inclusion criteria for interviewees were those with experience at both the SCH and the TCH, our study findings could result in bias. However, we feel that a certain amount of bias is required for accurately comparing the two sites. Regardless, in order to ensure generalizability, additional sites need to be studied and the findings replicated.

To establish potential future directions, suggestions were elicited for putting in place measures to reduce LOS. Social issues, such as financial barriers and support, were highlighted as factors that more specifically affected discharge at the TCH. The interventions suggested by interviewees attempted to efficiently mobilize healthcare resources to address the issue of LOS. This involved both screening in the preoperative setting for psychosocial barriers to discharge and patient prioritization for early discharge. Screening for psychosocial barriers to discharge could then facilitate referral to a multidisciplinary disposition clinic for personalized early discharge planning that can be in-person or virtual (26). These factors have been known to impact LOS following TKA (27, 28). In addition, prioritization of early discharge candidates for referring them to allied health teams will free up bed space in the orthopedics ward and ensure that arthroplasty patients receive specialized orthopedic PT care to accelerate recovery.

In conclusion, this study demonstrates that an SCH model is a safe and viable option for TKA patients operated upon by a large number of fellowship-trained arthroplasty surgeons. Such small hospitals may decrease LOS, while offering equivalent outcomes when compared with TCHs. However, further research must be conducted to confirm the presence of barriers leading to increased LOS and the interventions designed to address these barriers.

## Data Availability

The raw data supporting the conclusions of this article will be made available by the authors without undue reservation.

## Ottawa Arthroplasty Group

The members of this group are Geoffrey F. Dervin, Paul R. Kim, Robert J. Feibel, Hesham Abdelbary, Wade Gofton, George Grammatopoulos, and Paul E. Beaulé.

## Appendix A

**Appendix A:** Anesthesia Exclusion Criteria

Anesthesia Team at the Small Community Hospital suggests that ALL patients should be referred to another surgical center who meet any of the below criteria:
• Documented difficult airways • BMI 45 and over • Poor functional capacity (i.e. SOB or chest pain climbing one flight of stairs, or unable to do) • Poor or rapidly deteriorating state of physical health • Presence of pacemakers/defibrillators • Significant cardiac history (MI within 6 months, unstable angina, stages III-IV heart failure, uncontrolled dysrhythmias, mod-severe pulmonary hypertension, hypertrophic cardiomyopathy, severe mitral or aortic valve stenosis) • Significant lung disease (O2-dependent COPD, cystic fibrosis, severe asthma) • Significant hematologic disease (sickle cell anemia, thrombocytosis > 800, anemia of chronic disease with Hb < 100, factor deficiencies requiring pre-op administration, significant coagulopathy (i.e. hemophilia)) • Significant neurologic disease (myasthenia gravis, multiple sclerosis, or spinal cord disease with respiratory involvement) • Significant renal disease (ESRD, dialysis-dependent or with Cr >300) • Documented personal history of malignant hyperthermia
